# Multifunctional Liposomes Co-Modified with Ginsenoside Compound K and Hyaluronic Acid for Tumor-Targeted Therapy

**DOI:** 10.3390/polym16030405

**Published:** 2024-02-01

**Authors:** Xiaoyan You, Hui Liu, Yue Chen, Guoping Zhao

**Affiliations:** 1 College of Food and Bioengineering, Henan University of Science and Technology, Luoyang 471023, China; m18437956585@163.com; 2 Tianjin Institute of Industrial Biotechnology, Chinese Academy of Sciences, Tianjin 300308, China; 3 Haihe Laboratory of Synthetic Biology, Tianjin 300308, China; 4 Institute of Plant Physiology and Ecology, Chinese Academy of Sciences, Shanghai 200031, China

**Keywords:** liposomes, compound K, tumor-targeted therapy, PTX, hyaluronic acid

## Abstract

Liposomes show promise for anti-cancer drug delivery and tumor-targeted therapy. However, complex tumor microenvironments and the performance limitations of traditional liposomes restrict clinical translation. Hyaluronic acid (HA)-modified nanoliposomes effectively target CD44-overexpressing tumor cells. Combination therapy enhances treatment efficacy and delays drug resistance. Here, we developed paclitaxel (PTX) liposomes co-modified with ginsenoside compound K (CK) and HA using film dispersion. Compared to cholesterol (Ch), CK substantially improved encapsulation efficiency and stability. In vitro release studies revealed pH-responsive behavior, with slower release at pH 7.4 versus faster release at pH 5. In vitro cytotoxicity assays demonstrated that replacing Ch with CK in modified liposomes considerably decreased HCT-116 cell viability. Furthermore, flow cytometry and fluorescence microscopy showed a higher cellular uptake of PTX-CK-Lip-HA in CD44-high cells, reflected in the lower half maximal inhibitory concentrations. Overall, CK/HA-modified liposomes represent an innovative, targeted delivery system for enhanced tumor therapy via pH-triggered drug release and CD44 binding.

## 1. Introduction

Colorectal cancer is a common malignant tumor of the digestive system, and its incidence and mortality are still rapidly increasing in many medium and high human development index countries [1]. The chief clinical treatment methods for colorectal cancer include surgery, chemotherapy, radiotherapy, and targeted therapy. Chemotherapy remains the primary early treatment modality for colorectal and colon cancers. However, tumors typically exhibit heterogeneity, complexity, and instability, resulting in chemotherapy drug resistance and systemic toxicity, thereby limiting the clinical application of these drugs.

Nanoscale drug delivery systems have emerged as a highly promising avenue in cancer therapeutics by enhancing drug efficacy and the therapeutic index through the protection and stabilization of bioactive molecules [2]. As versatile nanocarriers, liposomes facilitate the transport of diverse hydrophobic and hydrophilic compounds [3,4]. Moreover, liposomes can amplify drug permeability and retention. The efficacy of drug delivery is further improved through targeted modifications during liposome preparation, which can concurrently mitigate toxicity and adverse effects [5]. Thus, liposomal-targeted therapy has shown promise in cancer treatment as one of the most efficacious co-carriers [6,7].

Despite their promise, liposomal-targeted therapies face limitations due to the intricate tumor microenvironment, insufficient tumor site accumulation, and non-specific harm to healthy cells/tissues, all of which detrimentally impact the therapeutic efficacy of liposomes in cancer treatment [5,8,9]. Concerns also persist regarding cholesterol (Ch), a fundamental component in lipid system formulations, due to its potential to elicit allergic reactions, activate the complement system, and induce cardiovascular side effects, including pulmonary hypertension [10,11].

Paclitaxel (PTX) is an essential anticancer agent used to treat various malignancies, including ovarian, breast, and colon cancers [12,13,14]. As a tetracyclic triterpene antitumor drug, PTX arrests the cell cycle in the G2/M phase by promoting microtubule polymerization, inhibiting degradation, and disrupting spindle formation during cytokinesis, culminating in cancer cell death. PTX elicits cytotoxicity in a concentration- and time-dependent manner [15]. Although PTX liposomal formulations are commercially available, suboptimal therapeutic efficacy necessitates improvements in carrier toxicity and stability [9,16]. Developing delivery systems that enhance liposomal stability and anti-proliferative activity could thus significantly improve their therapeutic impact.

Hyaluronic acid (HA) is a naturally occurring biocompatible, biodegradable, and non-toxic polysaccharide, widely applicable in drug delivery systems [17,18]. Abundant in bodily tissues, HA engages in diverse physiological functions through interactions with receptors and adhesion molecules. CD44, a cell membrane receptor, specifically binds to HA. HA-modified liposomes demonstrate pronounced affinity for CD44 receptors overexpressed on tumor cells, facilitating efficient uptake [19]. Consequently, HA modification prolongs drug circulation and enhances nanocarrier affinity to CD44-overexpressing tumor cells, increasing their growth-inhibitory effects [20,21]. The enhanced circulatory persistence of HA-modified drug delivery systems can be primarily attributed to their ability to evade the mononuclear phagocyte system through a hydrophilic barrier that minimizes opsonization, optimizing particle size to circumvent renal clearance, and altering biodistribution to preferentially target CD44 receptor-rich tissues, thereby reducing non-specific tissue distribution and extending systemic circulation [22,23,24].

Ginsenosides, the primary active constituents of *Panax ginseng*, bear a structural resemblance to cholesterol, allowing them to serve as natural cholesterol substitutes in liposomal formulations [25,26,27]. Compound K (CK), a potent ginsenoside derivative obtained from the deglycosylation of ginsenoside Rg3, exhibits superior biological activity compared to other ginsenosides, with neuroprotective [28], anti-inflammatory [29], and antidiabetic properties [30]. Studies have documented CK’s cytotoxic and growth-inhibitory effects on tumor cells alongside its potential to thwart tumor metastasis and proliferation [31,32,33]. However, CK’s limited solubility, suboptimal targeting, and severe adverse reactions significantly encumber its clinical efficacy [34].

Previous studies have illustrated that the synergistic application of dual-drug regimens can mitigate the limitations inherent to the administration of free drugs, simultaneously amplifying their cellular anti-cancer efficacy. Nonetheless, literature detailing the concurrent administration of paclitaxel and compound K (CK) remains sparse [14,27,35]. By harnessing the remarkable physicochemical attributes of hyaluronic acid (HA) and CK, our research has pioneered the development of a novel co-modified nanoliposomal formulation of paclitaxel (PTX), which integrates both CK and HA. This innovative approach exploits the cooperative interactions between HA and CK within the PTX liposomal lipid bilayer, resulting in a marked enhancement of both the nanoliposomes’ stability and their anti-cancer activity in vitro.

## 2. Materials and Methods

### 2.1. Materials

PTX was purchased from Aladdin Biochemical Technology Co., Ltd. (Shanghai, China). Lecithin from egg yolk was obtained from Macklin Biochemical Co., Ltd. (Shanghai, China). 3-(4,5-dimethylthiazol-2-yl)-2,5-diphenyltetrazolium bromide (MTT) assay kit, 4′,6-diamidino-2-phenylindole (DAPI), and coumarin 6 (C-6) were procured from Solarbio Science & Technology Co., Ltd. (Beijing, China). Trypsin, fetal bovine serum (FBS), DMEM medium, and penicillin-streptomycin mixtures were purchased from Gibco^®^ BRL (Carlsbad, CA, USA). CK was obtained from Yuanye Biotechnology Co., Ltd. (Shanghai, China). McCoy’s 5A medium was procured from Xiya Biochemical Technology Co., Ltd. (Tianjin, China).

### 2.2. Preparation of the Liposomes

In this study, PTX was used as the loaded drug. Three types of liposomes, namely, PTX-Ch-Lip, PTX-CK-Lip, and PTX-CK-Lip-HA, were prepared using the thin-film dispersion technique (Figure S1). These liposomes were meticulously crafted using a blend of egg lecithin, cholesterol (Ch), CK, and PTX. To clarify, the PTX-Ch-Lip formulation was synthesized by integrating egg lecithin, cholesterol, and paclitaxel (PTX) in a weight ratio of 100:30:1, respectively, with the abbreviation “Ch” indicating the incorporation of cholesterol. Initially, egg lecithin, cholesterol, and PTX were dissolved in chloroform in a flask. The flask was subjected to rotary evaporation at 48 °C under a vacuum of 200 mbar, which effectively removed the chloroform, resulting in the formation of a thin lipid layer. Subsequently, the dried lipid layer was rehydrated by adding 10 mL of phosphate buffer saline (PBS) at pH 7.4, followed by gentle spinning at 50 °C for 1 h. The suspension was then subjected to probing sonication using the JY92-IIDN sonicator (Ningbo Scientz Biotechnology Co., Ltd., Ningbo, China) until a semitransparent appearance was achieved. This sonication was performed in an ice bath for 10 min at 270 W with a sequence of 3 s of sonication followed by 5 s of rest, resulting in the formation of PTX-Ch-Lip. PTX-CK-Lip was obtained by replacing Ch with CK. Egg lecithin, CK, and PTX were added to the flask containing chloroform in a ratio of 100:30:1 by weight, where CK was dissolved in pure ethanol. Then, the PTX-CK-Lip solution was prepared in the same way. To fabricate the hyaluronic acid-modified liposomes, an equal volume of a 1% (*w*/*v*) HA solution was introduced dropwise to the preformed liposome formulation containing paclitaxel and compound K (PTX-CK-Lip). This mixture was then subjected to continuous stirring for 30 min at room temperature to ensure homogenous integration. The 1% HA solution was meticulously prepared by dissolving 0.4 mg of hyaluronic acid in 40 mL of ultrapure water. This solution was stirred overnight at room temperature to facilitate the complete dissolution of HA. Following the addition of HA, the surface of the PTX-CK-Lip mixture was fully coated with HA molecules, resulting in the formation of HA-modified liposomes, designated as PTX-CK-Lip-HA [36,37].

### 2.3. Transmission Electron Microscopy

The morphology and structure of the prepared liposomes were examined using transmission electron microscopy (TEM) with the HT-7800 microscope (Hitachi, Ltd., Tokyo, Japan), as previously described by Cui et al. [38]. In brief, 10 μL of the liposome solution was applied onto a carbon formvar-coated copper grid (300 mesh) and left undisturbed for 10 min, allowing the liposomes to attach to the grid. Then, the excess solution was gently removed with filter paper. Next, a 10 μL droplet of a 2% aqueous solution of phosphotungstic acid was applied to the copper grid and allowed to rest for 1 min. The excess liquid was then removed by gently blotting it with filter paper. Subsequently, the samples were air-dried before being observed and photographed under a transmission electron microscope at an accelerating voltage of 80 kV.

### 2.4. Size, Polydispersity Index, and Zeta Potential

The mean particle size, polydispersity index (PDI), and zeta potential of the three types of liposome suspensions were measured using a dynamic light scattering detector (Zetasizer, Nano ZS, Malvern Panalytical, Malvern, UK). In summary, 1 mL of each sample was taken and diluted tenfold with deionized water before measuring multiple samples of equal volume. The recorded results represent the average of three measurements.

### 2.5. The Encapsulation Efficiency of the Liposomes

The encapsulation efficiency of the liposomes was assessed using high-performance liquid chromatography (HPLC) with an ACQUITY Arc system (Waters, Milford, MA, USA) following a previously established method [39,40]. In brief, the amount of PTX encapsulated was determined by filtering out the precipitated PTX mixture through a 0.22 µm membrane filter. The encapsulated and total PTX liposome samples were then treated with methanol for 20 min to demulsify them. The total amount of PTX that was encapsulated was represented as W_A_, and the total amount of PTX was denoted as W_B_. The encapsulation efficiency (EE) of the liposomes was calculated using the following equation:

EE (%) = (W_A_/W_B_) × 100%

### 2.6. Fourier Transform Infrared Spectrum Analysis

Fourier transform infrared (FT-IR) spectroscopy (TCS SP5 II, Leica Microsystems, Wetzlar, Germany) was employed to investigate the differences in chemical bonding in the various types of liposome samples. Trehalose was added as a freeze-drying protective additive to the various liposome formulations, and the resulting samples were then freeze-dried using a vacuum freeze-drying mechanism to obtain a powdered form [41]. FT-IR spectra were recorded over the wavelength range of 4000–400 cm^−1^ and with 64 scans and a 4 cm^−1^ resolution against the background.

### 2.7. In Vitro Release Study

The drug-release curves of the three different types of liposomes were investigated using dialysis. Liposomes were exposed to PBS with 0.2% Tween 80 as a solubilizer to release the PTX solution. Conditions of pH 7.4 and pH 5.0 (phosphate buffer) were employed to mimic the conditions in normal blood circulation and the acidic microenvironment of tumor cells, respectively [42,43,44,45,46]. In brief, 1 mL of each sample was transferred into a prewashed dialysis bag (molecular weight cut off: 8000 Da, Solarbio Science & Technology Co., Ltd., Beijing, China) and securely sealed. The sealed end of the dialysis bag was then immersed in 30 mL of PBS (pH 7.4 and 5.0) at 37 °C while maintaining a magnetic stirring speed of 100 rpm. At predetermined time points (0.5, 1, 2, 3, 5, 6, 8, 10, and 12 h), 1 mL of each sample was withdrawn and replaced with an equal volume of fresh release medium. The concentration of PTX in each sample was determined using HPLC. Each sample experiment was performed in triplicates.

### 2.8. Storage Stability

The storage stability of the liposomes was assessed by measuring their EE [47]. In brief, the liposomal formulation was divided into two parts and stored at temperatures of 4 °C and 25 °C. Subsequently, samples were collected at various time intervals (1, 2, 3, 5, 7, 15, and 30 days), and the EE was determined using HPLC following the aforementioned method. The analysis of each sample was carried out in triplicates.

### 2.9. Cell Lines and Culture Conditions

The HCT-116 (human colorectal carcinoma) and MCF-7 (human breast adenocarcinoma) cell lines, both characterized by relatively high CD44 expression levels, were cultured in McCoy’s 5A and Dulbecco’s Modified Eagle’s Medium (DMEM), respectively. In contrast, the HepG2 cells (human hepatocellular carcinoma), exhibiting relatively lower CD44 expression, were maintained in DMEM [48,49,50]. All cell lines were incubated at 37 °C in a 5% CO_2_ humidified atmosphere. The basal medium was uniformly supplemented with 10% fetal bovine serum (FBS) and 1% antibiotic mixture (100 μg/mL streptomycin and 100 U/mL penicillin). Additionally, the MCF-7 culture medium was enriched with 1% non-essential amino acids to support the specific growth requirements. The cell lines used in this study were commercially obtained from Pricella Biotechnology Co., Ltd. (Wuhan, China). Upon attaining 80–90% confluence, the cells were detached with trypsin-EDTA, pelleted via centrifugation, and resuspended in fresh media for subsequent experimental use.

### 2.10. In Vitro Cytotoxicity Assays

The cells were seeded (5 × 10^3^ cells/well) into 96-well plates for 24 h. After achieving complete cell adherence, the culture medium was removed, and the drug intervention was performed, followed by observation. Subsequently, the HCT-116 cells were incubated with free PTX, free CK, PTX-Ch-Lip, PTX-CK-Lip, and PTX-CK-Lip-HA for 24 h. The MCF-7 and Hep G2 cells were incubated with PTX-CK-Lip and PTX-CK-Lip-HA for 24 h, respectively (as controls to validate the binding affinity of hyaluronic acid to CD44). After incubation, the drug formulations were discarded, and the MTT assay was carried out in accordance with the kit’s protocol. The OD value of each well was measured using a microplate reader (Infinite 200 PRO, TECAN, Männedorf, Switzerland) at 490 nm. The cell inhibition rate and IC50 were calculated using GraphPad Prism 8.0 software. The MTT results were expressed as the mean ± standard deviation (SD) of three replicates. The cell inhibition rate was calculated using the following equation:

$$\mathrm{Cell}\;\mathrm{inhibition}\;\left(\%\right)=\left(1-\frac{\mathrm{Absorbance}\;\left(\mathrm{test}\right)}{\mathrm{Absorbance}\;\left(\mathrm{control}\right)}\right)\times 100\;\%$$

### 2.11. Cellular Uptake Assay

The cellular uptake of three types of liposomes in cancer cells with different CD44 expression levels was explored using flow cytometry and inverted fluorescence microscopy. In brief, different cells were seeded at a density of 2 × 10^5^ cells/well into 6-well plates and cultured for 24 h until the cells were fully adhered. Following incubation, coumarin 6 (C-6)-labeled liposomes (incorporated into the hydrophobic layer of the liposomes via substituent binding and cross-linking) were added to 6-well plates at a final concentration of 500 ng/mL. The plates were then incubated for 4 h and subsequently washed three times with ice-cold PBS. After being washed, the cells were fixed with 4% paraformaldehyde for 10 min at room temperature. The plates were then washed three times with ice-cold PBS, and DAPI (10 μg/mL) was added to stain the cells. After 10 min, the DAPI was removed, and the 6-well plates were washed with ice-cold PBS three times. An inverted fluorescence microscope (IX73, Olympus, Japan) was used to visualize the fluorescence of C-6 in the cells.

All three cancer cell types were seeded at a density of 1 × 10^5^ cells/well into 6-well plates and cultured for 24 h until the cells were fully adhered. Then, the medium was removed, and the test samples [including C-6-labeled PTX-CK-Lip and PTX-CK-Lip-HA (PTX concentration of 0.5 µg/mL), blank sample, C-6 sample (single-positive sample)] were added to the 6-well plates for 4 h in an incubator at 37 °C with 5% CO_2_. Subsequently, the samples were washed three times with ice-cold PBS, detached with trypsin, and centrifuged. Finally, the cells were resuspended in 500 μL of PBS and stored on ice until analysis occurred. A total of 10,000 cells were interrogated from each sample using flow cytometry (BD Fortessa X-20, BD Biosciences, Houston, TX, USA), and the mean fluorescence intensity of the samples were tested. The percentage of these cells were subsequently analyzed using FlowJo v10.8.1 [51]. The relative cellular uptake rate was calculated using the following formula (F_A_ denotes the mean fluorescence intensity of the test sample or blank sample, and F_B_ denotes the mean fluorescence intensity of the C-6 sample).

The relative cellular uptake (%) = F_A_/F_B_ × 100%

### 2.12. Statistical Analysis

The data were expressed as mean ± SD. The data and graphs were generated using GraphPad Prism 8.0. The mean values were compared using the unpaired *t*-test, with a significance level set at 0.05 (*p* < 0.05). All measurements were performed in triplicates.

## 3. Results

### 3.1. Size, PDI, Zeta Potential, and EE

**Figure 1:**
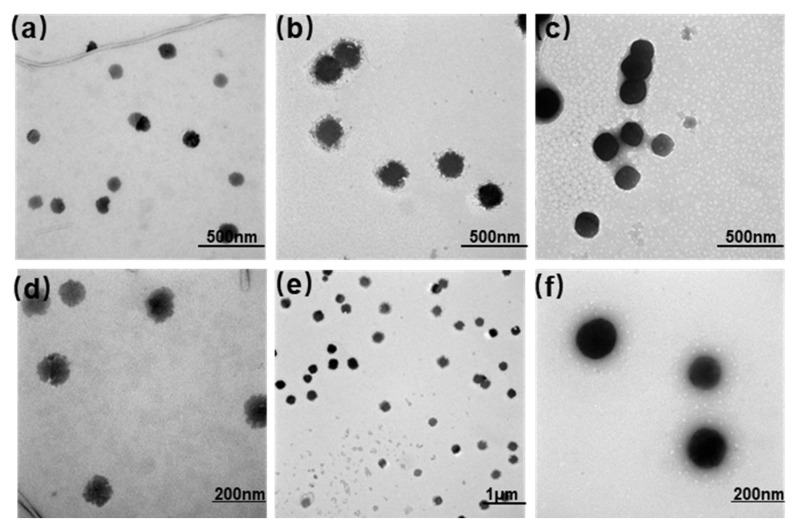
TEM images of (**a**,**d**) PTX-Ch-Lip, (**b**,**e**) PTX-CK-Lip, and (**c**,**f**) PTX-CK-Lip-HA, where abc has a resolution of ×15.0 k, e has a resolution of ×5.0 k, and df has a resolution of ×30.0 k.

This study prepared three types of liposomes (PTX-Ch-Lip, PTX-CK-Lip, and PTX-CK-Lip-HA) using the thin-film hydration method. The particle size and zeta potential of the liposomes were measured using a dynamic light scattering detector and are shown in Table 1. The particle size of liposomes plays a crucial role in determining the capacity, release, and bioavailability of the encapsulated material. As shown in Table 1, there was no significant difference in the particle size between PTX-Ch-Lip and PTX-CK-Lip. Following the addition of HA, the commodified liposomes increased by approximately 20 nm. These results provide preliminary evidence for the successful attachment of HA to the surface of liposomes. The TEM results (Figure 1) confirm the spherical shape of the liposomes with smooth surfaces, suggesting that the addition of hyaluronic acid and CK did not disrupt the morphology of the liposomes. The particle size of the liposomes increased upon the addition of HA, which is consistent with the results obtained from particle size analyses. Furthermore, PTX-CK-Lip and PTX-CK-Lip-HA exhibited significantly improved EE values than PTX-Ch-Lip.

### 3.2. FT-IR Spectroscopy

**Figure 2:**
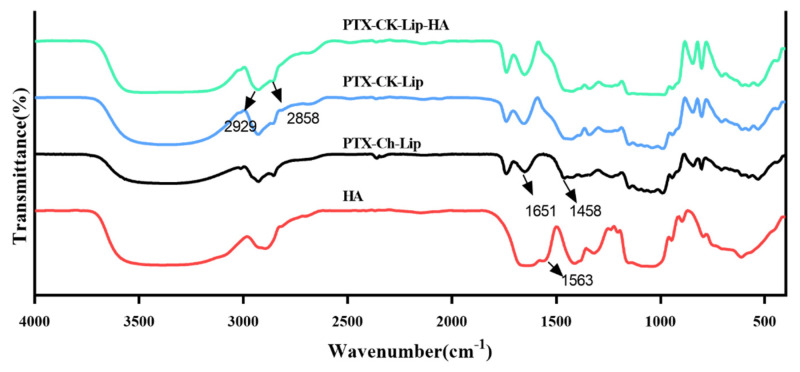
FT-IR spectra of pure HA, PTX-CK-Lip, PTX-CK-Lip-HA, and PTX-Ch-Lip.

The potential interactions between HA, CK, and the liposome bilayer were investigated using FT-IR spectroscopy. The FT-IR spectra of pure HA, PTX-Ch-Lip, PTX-CK-Lip, and PTX-CK-Lip-HA are shown in Figure 2. The characteristic peaks of cholesterol attributable to the double bond (C=C) in the second ring are observed at 1651 cm^−1^. The band at 1458 cm^−1^ belongs to the asymmetric tensile vibration of the –CH_2_– and –CH_3_ groups [52]. Compared with PTX-Ch-Lip, PTX-CK-Lip exhibited enhanced peaks at 1651 and 1458 cm^−1^, indicating the replacement of cholesterol with CK. The main absorption peak of HA at 1563 cm^−1^ is caused by the stretching vibrations of C=O in the COO– group. In the infrared spectra of PTX-CK-Lip and PTX-CK-Lip-HA, the absorption peaks at 2929 and 2858 cm^−1^ are attributable to the vibrations of the hydrophobic –CH_2_– group within the phospholipid molecular layer. The positions of these two peaks remained unchanged in both types of liposomes, suggesting that the modification with HA did not alter the internal structure of the liposomes. After the attachment of liposomes with HA, the characteristic absorption peak of HA vanished, suggesting the absence of stretching vibrations of the COO– group in the HA solution. The observed phenomenon may be attributed to the fact that HA is modified to the surface of liposomes, which is consistent with previous scanning electron microscopy results. Additionally, the FT-IR results validated the successful adhesion of HA to the liposome nanoparticles, further supporting previous analysis results.

### 3.3. In Vitro Drug Release

**Figure 3:**
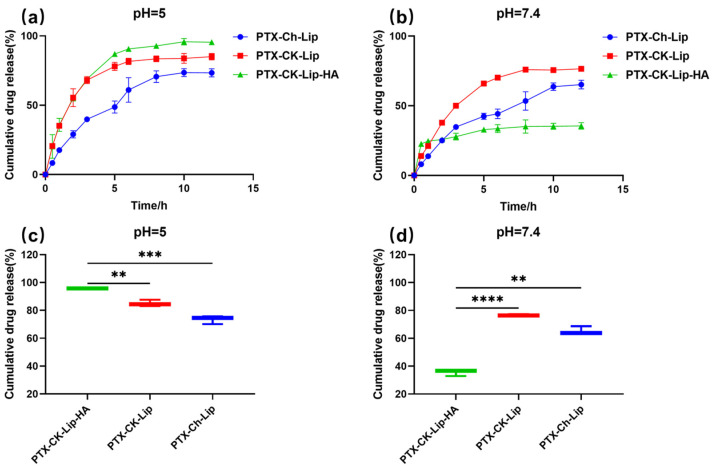
The cumulative drug release of liposomes in (**a**) pH = 5.0 and (**b**) pH = 7.4 PBS at 37 °C from 0 to 12 h. The cumulative drug release of liposomes in (**c**) pH = 5.0 and (**d**) pH = 7.4 PBS at 37 °C at 12 h. (*n* = 3; mean ± SD), ** *p* < 0.01, *** *p* < 0.001, **** *p* < 0.0001.

The drug-release properties of the liposomes were investigated using the dialysis method under various pH conditions. The release rates of the liposomes from the release matrices under different pH conditions are shown in Figure 3. Overall, all three types of liposomes exhibited rapid release within the first 5 hours, followed by a slower release from the 5th to the 10th hour, ultimately stabilizing at the 12th hour. Additionally, the release rate of PTX-CK-Lip was higher than that of PTX-Ch-Lip at both pH 7.4 and pH 5. Notably, PTX-CK-Lip-HA demonstrated a better release profile compared to PTX-Ch-Lip. Under the condition of pH 7.4, the cumulative release rates of PTX-Ch-Lip and PTX-CK-Lip-HA were 65% and 35%, respectively. The incorporation of HA substantially decreased PTX release from the liposomes when they were surrounded by HA. This effect may be attributed to HA’s strong hydrophilic nature, which enables it to rapidly absorb water. Consequently, HA forms a dense hydrated film around the liposomes, which, in turn, reduces the fluidity and permeability of the phospholipid bilayer, thereby hindering drug release [53]. Conversely, when subjected to pH 5.0 conditions, the release rate of PTX-CK-Lip-HA significantly increased, resulting in a release percentage of 95%. This phenomenon can be attributed to the fact that HA-modified liposomes exhibit instability in acidic aqueous solutions and are susceptible to hydrolysis, leading to the random breakage of polymer chains, which increases the fluidity and permeability of the phospholipid bilayer, leading to an incomplete liposome structure and increase in drug release from the liposomes [17]. This is consistent with previous results that the drug-release behavior of HA-modified liposomes exhibits significant pH dependence [54,55]. It may be indicated that before PTX-CK-Lip-HA reached the tumor site, it was able to maintain the structural integrity as much as possible under normal physiological conditions to reduce the leakage of the drug, thus reducing the damage to normal cells [49]. Moreover, the inhibitory effect of liposomes on tumor cells is enhanced by targeting to enrich as many drugs as possible to the tumor cells and then releasing them rapidly [49]. Consequently, the use of the newly prepared PTX-CK-Lip-HA formulation inhibits the growth of tumor cells while reducing toxicity and associated side effects to normal cells [42].

### 3.4. Stability Study

**Figure 4:**
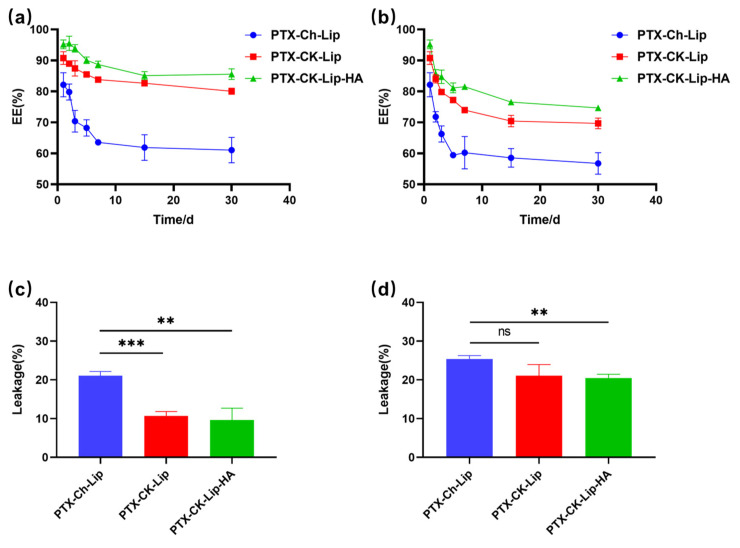
The storage stability of liposome formulations at (**a**) 4 °C and (**b**) 25 °C within 30 days. The leakage of liposome formulations at (**c**) 4 °C and (**d**) 25 °C on the 30^th^ day. (*n* = 3; mean ± SD), ** *p* < 0.01, *** *p* < 0.001.

The stability of the liposomes under varying temperature conditions is crucial for protecting the drug from external environmental factors. As shown in Figure 4, PTX-CK-Lip exhibited a slower drug leakage rate compared to PTX-Ch-Lip, indicating that PTX-CK-Lip increased liposome stability. This effect may be attributed to the addition of ginsenosides, which altered the size and morphology of the liposomes, affecting the order of lipid molecules and membrane fluidity [26]. The reduction in the leakage rate of PTX-CK-Lip-HA compared to PTX-CK-Lip may be due to the hydrophilic HA in the outer layer of the liposomes forming a protective barrier that prevents the accumulation of liposomes and renders them more stable [53]. This observation suggests that PTX-CK-Lip-HA has excellent drug protection ability even under different temperature conditions. Specifically, the leakage rate of PTX-CK-Lip-HA liposomes at 4 °C was consistently lower than at 25 °C, indicating greater stability at the lower temperature. 

### 3.5. In Vitro Cytotoxicity

**Figure 5:**
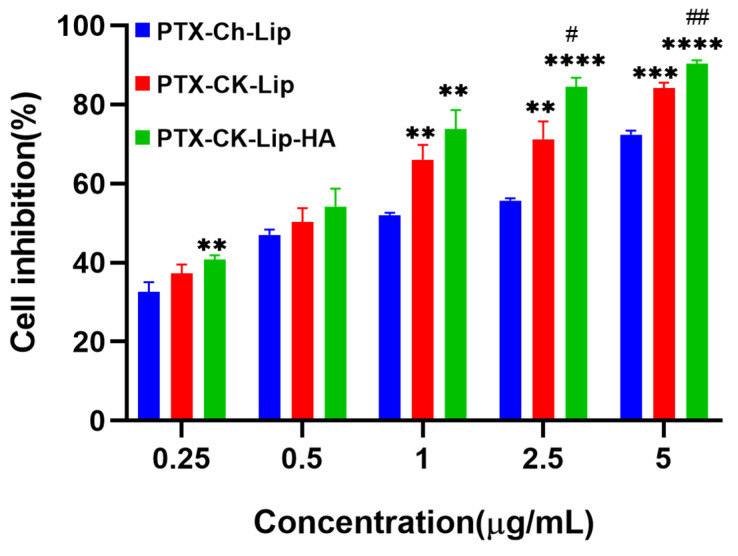
Cell inhibition rates of various formulations on HCT-116 cells treated with PTX-Ch-Lip, PTX-CK-Lip, and PTX-CK-Lip-HA. ** *p* < 0.01 vs. PTX-Ch-Lip; *** *p* < 0.001 vs. PTX-Ch-Lip; **** *p* < 0.0001 vs. PTX-Ch-Lip; ^#^ *p* < 0.05 vs. PTX-CK-Lip and ^##^ *p* < 0.01 vs. PTX-CK-Lip.

To elucidate the cytotoxic impacts of co-modified liposomes on HCT-116 colorectal carcinoma cells, an MTT assay was employed. Figure 5 illustrates the results following the 24-h exposure of the cells to various liposomal formulations. A comparative analysis among three distinct liposomal variants was conducted to assess their antineoplastic efficacy. The data revealed that the liposomes exerted a dose-dependent inhibitory effect on HCT-116 cell proliferation, with notable activity commencing at concentrations exceeding 1 µg/mL (PTX-Ch-Lip and PTX-CK-Lip-HA). Specifically, the PTX-Ch-Lip formulation at 1 μg/mL resulted in a minimal inhibition rate of 52.05%. Conversely, HCT-116 cells treated with PTX-CK-Lip and PTX-CK-Lip-HA at equivalent PTX concentrations demonstrated cell inhibition rates of 66.07% and 73.77%, respectively. These findings suggest that the antitumor potency of PTX is significantly enhanced with the integration of CK within the liposomal structure, indicative of a synergistic interaction between CK and PTX. Moreover, the hyaluronic acid (HA)-coated PTX-CK-Lip mixture (denoted as PTX-CK-Lip-HA) exhibited a more pronounced antitumor effect compared to its uncoated counterpart, an effect that was amplified with increasing concentration. This enhanced efficacy can be attributed to the high affinity of HA for the cell surface receptor CD44, which facilitates the targeted accumulation of PTX-CK-Lip-HA at the HCT-116 cells’ site and promotes cellular uptake, ultimately augmenting the therapeutic impact in vitro.

Table 2 presents data demonstrating that PTX-CK-Lip has significantly lower IC50 values compared to free CK, suggesting that encapsulating CK within liposomes enhances its ability to inhibit the growth of HCT-116 cancer cells. Moreover, PTX-CK-Lip exhibited a more potent inhibitory effect on HCT-116 cells than PTX-Ch-Lip, as evidenced by the statistically significant differences in their IC50 values (PTX-CK-Lip: 0.48 ± 0.03; PTX-Ch-Lip: 0.91 ± 0.02, **** *p* < 0.0001). These findings imply that CK may exert a synergistic effect when combined with PTX in liposomal formulations. Upon the incorporation of hyaluronic acid (HA) into PTX-CK-Lip, a notable reduction in the IC50 value was observed (PTX-CK-Lip: 0.48 ± 0.03; PTX-CK-Lip-HA: 0.38 ± 0.04, * *p* < 0.05), indicating an enhanced inhibitory effect on HCT-116 tumor cells. The modification of liposomal surfaces with HA appears to significantly augment the suppression of CD44-overexpressing cancer cells. In summary, the synergistic inclusion of CK and HA has been demonstrated to substantially amplify the inhibitory impact on HCT-116 cell proliferation.

### 3.6. Cellular Uptake

**Figure 6:**
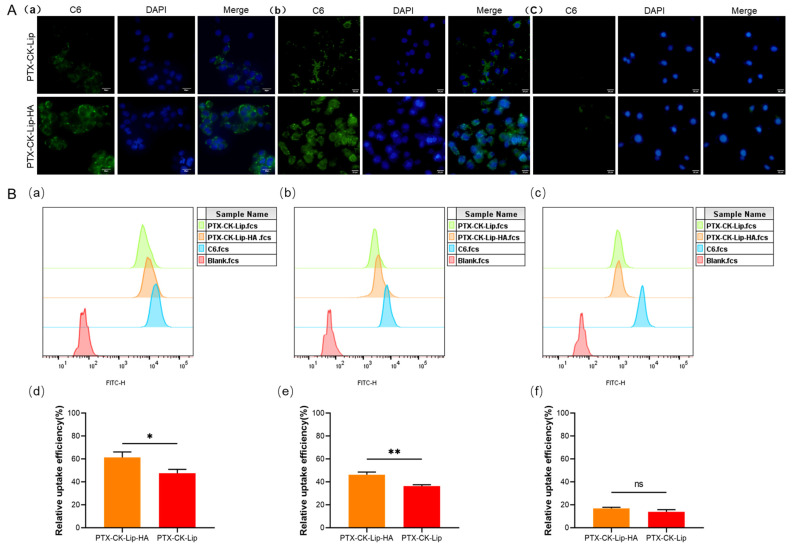
Cellular uptake assay of liposomes. (**A**) Inverted fluorescence microscopy images of C6-labeled PTX-CK-Lip and PTX-CK-Lip-HA in (**a**) HCT-116 cells, (**b**) MCF-7 cells, and (**c**) Hep G2 cells (scale bar = 25μm). (**B**) FCM of C6-labeled PTX-CK-Lip and PTX-CK-Lip-HA in (**a**) HCT-116 cells, (**b**) MCF-7 cells, and (**c**) Hep G2 cells. The relative cellular uptake efficiency rate of PTX-CK-Lip and PTX-CK-Lip-HA in (**d**) HCT-116 cells, (**e**) MCF-7 cells, and (**f**) Hep G2 cells. (*n* = 3; mean ± SD), * *p* < 0.05, ** *p* < 0.01.

To investigate the cellular uptake of PTX-CK-Lip-HA, all three cell types with varying levels of surface CD44 expression were utilized. As shown in Figure 6A, the fluorescence intensity of C-6-labeled PTX-CK-Lip-HA was significantly higher than that of PTX-CK-Lip in HCT-116 and MCF-7 cells with relatively high CD44 expression. There was no significant difference in fluorescence intensity between the two liposomes in HepG2 cells with low CD44 expression [50]. The IC50 values of the liposomes in MCF-7 and HepG2, as shown in Table 2, indirectly validate the influence of varied CD44 expression levels on liposome uptake by the cells. This observation can be attributed to the ability of HA-coated liposomes to specifically target the CD44 receptor protein to enhance the cellular uptake efficiency by receptor-ligand interactions.

Flow cytometry (FCM) is commonly used to quantitatively analyze the in vitro uptake of cells. The results of the FCM are shown in Figure 6B. In MCF-7 and HCT-116 cells with high expression levels of CD44, PTX-CK-Lip-HA exhibited a significantly higher mean fluorescence intensity compared to PTX-CK-Lip. There was no significant difference in the mean fluorescence intensity between PTX-CK-Lip and PTX-CK-Lip-HA in Hep G2 cells with low expression of CD44. This point is further confirmed by their IC50 value. The reason for this result may be that in cells with high CD44 expression, specific binding of CD44 to HA increases the relative uptake efficiency of the HA-liposome. These results are consistent with those of inverted fluorescence microscopy and cell viability studies.

## 4. Discussion

Colorectal cancer (CRC) ranks as the third most prevalent malignancy and the fourth leading cause of cancer-related mortality worldwide [56,57,58,59,60]. Projections indicate that by 2040, the global incidence of CRC may reach approximately 3.2 million cases, with the highest numbers anticipated in China and the United States [61]. Chemotherapy remains the cornerstone of early-stage treatment for colorectal and colon cancers; however, the intrinsic heterogeneity, complexity, and instability of tumors often lead to chemotherapy resistance and systemic toxicity, curtailing the clinical efficacy of these treatments. Liposomes as drug delivery vehicles are capable of encapsulating a broad spectrum of hydrophobic and hydrophilic agents, thereby enhancing drug permeability and retention. Through targeted modifications in their preparation, liposomes can be optimized to improve delivery efficacy while simultaneously mitigating toxicity and adverse effects, positioning liposomal-targeted therapy as a notably promising and effective strategy in cancer treatment [3,4,5,6,7]. In the current study, we synthesized a series of paclitaxel (PTX)-loaded liposomes—PTX-Ch-Lip, PTX-CK-Lip, and PTX-CK-Lip-HA—to evaluate their antitumor efficacy against HCT-116 cells. The results suggest that liposomes modified with ginsenoside compound K (CK) and hyaluronic acid (HA) could serve as an innovative, targeted delivery systems, enhancing tumor therapy effectiveness through pH-triggered drug release and CD44 receptor binding.

Research indicates that nanoparticle size is a crucial determinant of drug transport efficiency [62]. The liposomes developed in this study demonstrated uniform particle size, with a polydispersity index (PDI) of 0.28 ± 0.01 and an average size of 188.5 ± 0.40 nm, which is less than 200 nm [63]. This smaller size allows the liposomes to evade detection by the reticuloendothelial system. During in vitro characterization, PTX-CK-Lip-HA exhibited superior encapsulation efficiency (EE) and stability compared to PTX-Ch-Lip and PTX-CK-Lip, likely due to the protective effects of the incorporated HA and CK [26]. In cytotoxicity assays, PTX-CK-Lip-HA demonstrated a more potent inhibitory effect on cancer cells, potentially due to unique mechanisms facilitating cellular drug uptake. Previous studies have identified CD44-mediated endocytosis as a key mechanism for the internalization of HA-targeted nanocarriers [64]. Once internalized, the drug is recognized and subsequently eliminated [53]. PTX-CK-Lip-HA is actively targeted and internalized via endocytosis, with HA-modified liposomes showing a propensity for binding to the CD44 receptor, which is overexpressed on the surfaces of HCT-116 and MCF-7 cells, thereby enhancing cellular uptake [65,66]. In contrast, cells with lower CD44 expression levels exhibit reduced uptake. Flow cytometry (FCM) was utilized to quantify the relative fluorescence intensity, confirming the increased uptake of PTX-CK-Lip-HA in cells with higher CD44 expression. While energy-dependent endocytosis has been identified as the primary pathway for uptake, a detailed understanding of the specific mechanisms and intracellular trafficking routes requires further elucidation. The elevated uptake of PTX-CK-Lip-HA could also result from variations in cell membrane fusion or endocytosis efficiency, which warrants additional investigation [67].

The high uptake rate of co-delivered nanoparticles helps to reduce the damage of drugs to normal cells during chemotherapy and prolongs their in vivo action by improving the stability of the carriers, which is conducive to the enhancement of the accumulation of anticancer drugs in the tumors and the increase in their bioavailability [35]. Co-modified liposomes have enhanced retention and permeability (EPR) effects in tumor cells, and releasing the drug at pH 5.0 attenuates the side effects on normal cells [49], which allows liposomes to preferentially accumulate in tumor tissue due to its leaky vasculature and impaired lymphatic drainage. Upon reaching the acidic tumor site, the liposomes are triggered to release their payload more rapidly due to the pH-sensitive properties of their coating, thereby concentrating the drug’s action within the tumor and reducing systemic exposure. The effects of PTX-loaded liposomes co-modified with HA and CK on HCT116 have not been previously investigated, and this study verified its potential therapeutic effect on HCT-116. While our study establishes a foundational understanding, subsequent research endeavors must concentrate on delineating the precise cellular uptake mechanisms. Moreover, it is imperative to authenticate the anti-proliferative efficacy of these liposomes through rigorous in vivo animal studies. In addition, the exploration of their clinical applicability warrants further examination.

## 5. Conclusions

In conclusion, the modification of liposome surfaces with CK and HA resulted in a significant enhancement in their capacity to suppress the growth of colon cancer cells. The liposome exhibits an EE of 95% along with excellent stability and pH-responsive characteristics, which are essential for enhancing the synergistic anti-proliferative effect of multiple free drugs and their targeted drug delivery. The synergistic antitumor cellular effect of CK and the active targeting effect of HA were evaluated by calculating cytotoxicity, comparing IC50 values, and conducting cellular uptake experiments on various cell lines. As a novel liposomal formulation, PTX-CK-Lip-HA may be a potential therapeutic approach for the treatment of colon cancer, and thus, this system can be further investigated.

## Figures and Tables

**Table 1:** Characterization of different liposomes (*n* = 3, mean ± SD).

Sample	Size (nm)	PDI	Zeta Potential (mV)	EE (%)
PTX-Ch-Lip	162.40 ± 1.50	0.24 ± 0.00	−6.85 ± 0.48	76.94 ± 0.04
PTX-CK-Lip	164.00 ± 1.90	0.27 ± 0.00	−5.82 ± 0.15	90.78 ± 2.05
PTX-CK-Lip-HA	188.50 ± 0.40	0.28 ± 0.01	−9.00 ± 0.48	95.31 ± 1.41

**Table 2:** IC50 values of different liposomes in different cells.

Cell Line	Sample	IC50 (µg/mL)
HCT-116	Free CK	33.83 ± 2.27
	Free PTX	1.38 ± 0.09
	PTX-Ch-Lip	0.91 ± 0.02
	PTX-CK-Lip	0.48 ± 0.03
	PTX-CK-Lip-HA	0.38 ± 0.04
MCF-7	PTX-CK-Lip	1.35 ± 0.02
	PTX-CK-Lip-HA	0.37 ± 0.05
Hep G2	PTX-CK-Lip	1.38 ± 0.17
	PTX-CK-Lip-HA	1.29 ± 0.17

## Data Availability

Data are contained within the article.

## Supplementary Materials

The following supporting information can be downloaded at: https://www.mdpi.com/article/10.3390/polym16030405/s1, Figure S1: Basic structures of a liposome (a) PTX-Ch-Lip, (b) PTX-CK-Lip, and (c) PTX-CK-Lip-HA and Project info 1 of IC50 calculation methods.

## References

[1] Arnold M., Sierra M.S., Laversanne M., Soerjomataram I., Jemal A., Bray F. (2017). Global patterns and trends in colorectal cancer incidence and mortality. Gut.

[2] Cardoso R.V., Pereira P.R., Freitas C.S., Paschoalin V.M.F. (2022). Trends in Drug Delivery Systems for Natural Bioactive Molecules to Treat Health Disorders: The Importance of Nano-Liposomes. Pharmaceutics.

[3] Zahednezhad F., Saadat M., Valizadeh H., Zakeri-Milani P., Baradaran B. (2019). Liposome and immune system interplay: Challenges and potentials. J. Control. Release.

[4] Nikolova M.P., Kumar E.M., Chavali M.S. (2022). Updates on Responsive Drug Delivery Based on Liposome Vehicles for Cancer Treatment. Pharmaceutics.

[5] Yang B., Song B.-P., Shankar S., Guller A., Deng W. (2021). Recent advances in liposome formulations for breast cancer therapeutics. Cell. Mol. Life Sci..

[6] Sang R., Stratton B., Engel A., Deng W. (2021). Liposome technologies towards colorectal cancer therapeutics. Acta Biomater..

[7] Riaz M.K., Riaz M.A., Zhang X., Lin C., Wong K.H., Chen X., Zhang G., Lu A., Yang Z. (2018). Surface Functionalization and Targeting Strategies of Liposomes in Solid Tumor Therapy: A Review. Int. J. Mol. Sci..

[8] Amiryaghoubi N., Fathi M., Barar J., Omidian H., Omidi Y. (2023). Advanced nanoscale drug delivery systems for bone cancer therapy. Biochim. Biophys. Acta (BBA)-Mol. Basis Dis..

[9] Pei Q., Jiang B., Hao D., Xie Z. (2023). Self-assembled nanoformulations of paclitaxel for enhanced cancer theranostics. Acta Pharm. Sin. B.

[10] Wang G.-F., Guan L.-H., Zhou D.-X., Chen D.-D., Zhang X.-C., Ge J.-B. (2020). Serum High-Density Lipoprotein Cholesterol is Significantly Associated with the Presence and Severity of Pulmonary Arterial Hypertension: A Retrospective Cross-Sectional Study. Adv. Ther..

[11] Niyonzima N., Halvorsen B., Sporsheim B., Garred P., Aukrust P., Mollnes T.E., Espevik T. (2017). Complement activation by cholesterol crystals triggers a subsequent cytokine response. Mol. Immunol..

[12] Chen W.-C., Huang H.-J., Chang T.-C., Chou H.-H. (2020). Dose-dense chemotherapy with weekly paclitaxel and 3-weekly carboplatin for recurrent ovarian cancer. Taiwan. J. Obstet. Gynecol..

[13] Shen J., Chen C., Li Z., Hu S. (2020). Paclitaxel Promotes Tumor-Infiltrating Macrophages in Breast Cancer. Technol. Cancer Res. Treat..

[14] Pham D.T., Saelim N., Tiyaboonchai W. (2020). Paclitaxel loaded EDC-crosslinked fibroin nanoparticles: A potential approach for colon cancer treatment. Drug Deliv. Transl. Res..

[15] Ahmed Khalil A., Rauf A., Alhumaydhi F.A., Aljohani A.S., Javed M.S., Khan M.A., Khan I.A., El-Esawi M.A., Bawazeer S., Bouyahya A. (2022). Recent Developments and Anticancer Therapeutics of Paclitaxel: An Update. Curr. Pharm. Des..

[16] Xu Q., Trissel L.A., Martinez J.F. (1994). Stability of paclitaxel in 5% dextrose injection or 0.9% sodium chloride injection at 4, 22, or 32 degrees C. Am. J. Hosp. Pharm..

[17] Song M., Liang Y., Li K., Zhang J., Zhang N., Tian B., Han J. (2019). Hyaluronic acid modified liposomes for targeted delivery of doxorubicin and paclitaxel to CD44 overexpressing tumor cells with improved dual-drugs synergistic effect. J. Drug Deliv. Sci. Technol..

[18] Fu C.-P., Cai X.-Y., Chen S.-L., Yu H.-W., Fang Y., Feng X.-C., Zhang L.-M., Li C.-Y. (2023). Hyaluronic Acid-Based Nanocarriers for Anticancer Drug Delivery. Polymers.

[19] Sanità G., Carrese B., Lamberti A. (2020). Nanoparticle Surface Functionalization: How to Improve Biocompatibility and Cellular Internalization. Front. Mol. Biosci..

[20] Wang J., Liu D., Guan S., Zhu W., Fan L., Zhang Q., Cai D. (2020). Hyaluronic acid-modified liposomal honokiol nanocarrier: Enhance anti-metastasis and antitumor efficacy against breast cancer. Carbohydr. Polym..

[21] Salari N., Mansouri K., Valipour E., Abam F., Jaymand M., Rasoulpoor S., Dokaneheifard S., Mohammadi M. (2021). Hyaluronic acid-based drug nanocarriers as a novel drug delivery system for cancer chemotherapy: A systematic review. DARU J. Pharm. Sci..

[22] Dosio F., Arpicco S., Stella B., Fattal E. (2016). Hyaluronic acid for anticancer drug and nucleic acid delivery. Adv. Drug Deliver. Rev..

[23] Kenchegowda M., Rahamathulla M., Hani U., Begum M.Y., Guruswamy S., Osmani R.A.M., Gowrav M.P., Alshehri S., Ghoneim M.M., Alshlowi A. (2022). Smart Nanocarriers as an Emerging Platform for Cancer Therapy: A Review. Molecules.

[24] Choi K.Y., Min K.H., Yoon H.Y., Kim K., Park J.H., Kwon I.C., Choi K., Jeong S.Y. (2011). PEGylation of hyaluronic acid nanoparticles improves tumor targetability. Biomaterials.

[25] Wang H., Zheng Y., Sun Q., Zhang Z., Zhao M., Peng C., Shi S. (2021). Ginsenosides emerging as both bifunctional drugs and nanocarriers for enhanced antitumor therapies. J. Nanobiotechnology.

[26] Hong C., Wang D., Liang J., Guo Y., Zhu Y., Xia J., Qin J., Zhan H., Wang J. (2019). Novel ginsenoside-based multifunctional liposomal delivery system for combination therapy of gastric cancer. Theranostics.

[27] Zhu Y., Liang J., Gao C., Wang A., Xia J., Hong C., Zhong Z., Zuo Z., Kim J., Ren H. (2021). Multifunctional ginsenoside Rg3-based liposomes for glioma targeting therapy. J. Control. Release.

[28] Seo J.Y., Ju S.H., Oh J., Lee S.K., Kim J.-S. (2016). Neuroprotective and Cognition-Enhancing Effects of Compound K Isolated from Red Ginseng. J. Agric. Food Chem..

[29] Kim E., Yi Y.-S., Son Y.-J., Han S.Y., Kim D.H., Nam G., Hossain M.A., Kim J.-H., Park J., Cho J.Y. (2018). BIOGF1K, a compound K-rich fraction of ginseng, plays an antiinflammatory role by targeting an activator protein-1 signaling pathway in RAW264.7 macrophage-like cells. J. Ginseng Res..

[30] Wei S., Li W., Yu Y., Yao F., Lixiang A., Lan X., Guan F., Zhang M., Chen L. (2015). Ginsenoside Compound K suppresses the hepatic gluconeogenesis via activating adenosine-5′monophosphate kinase: A study in vitro and in vivo. Life Sci..

[31] Zhang B., Fu R., Duan Z., Shen S., Zhu C., Fan D. (2022). Ginsenoside CK induces apoptosis in triple-negative breast cancer cells by targeting glutamine metabolism. Biochem. Pharmacol..

[32] Huang J., Pan D., Liu F., Hong Y., Huang G., Huang X., Wang X., Lin Z. (2022). Ginsenoside compound K inhibits the proliferation, migration and invasion of Eca109 cell via VEGF-A/Pi3k/Akt pathway. J. Cardiothorac. Surg..

[33] Xue Z., Fu R., Duan Z., Chi L., Zhu C., Fan D. (2021). Inhibitory Effect of pH-Responsive Nanogel Encapsulating Ginsenoside CK against Lung Cancer. Polymers.

[34] Yan C., Shi W., Gu J., Lee R.J., Zhang Y. (2021). Design of a Novel Nucleus-Targeted NLS-KALA-SA Nanocarrier to Delivery Poorly Water-Soluble Anti-Tumor Drug for Lung Cancer Treatment. J. Pharm. Sci..

[35] Gong J., Shi T., Liu J., Pei Z., Liu J., Ren X., Li F., Qiu F. (2023). Dual-drug codelivery nanosystems: An emerging approach for overcoming cancer multidrug resistance. Biomed. Pharmacother..

[36] Li J., Wang X., Zhang T., Wang C., Huang Z., Luo X., Deng Y. (2015). A review on phospholipids and their main applications in drug delivery systems. Asian J. Pharm. Sci..

[37] Zhao F., Li R., Liu Y., Chen H. (2023). Perspectives on lecithin from egg yolk: Extraction, physicochemical properties, modification, and applications. Front. Nutr..

[38] Cui T., Jia A., Yao M., Zhang M., Sun C., Shi Y., Liu X., Sun J., Liu C. (2021). Characterization and Caco-2 Cell Transport Assay of Chito-Oligosaccharides Nano-Liposomes Based on Layer-by-Layer Coated. Molecules.

[39] Hong C., Liang J., Xia J., Zhu Y., Guo Y., Wang A., Lu C., Ren H., Chen C., Li S. (2020). One Stone Four Birds: A Novel Liposomal Delivery System Multi-functionalized with Ginsenoside Rh2 for Tumor Targeting Therapy. Nano-Micro Lett..

[40] Lim S.-J., Hong S.-S., Choi J.Y., Kim J.O., Lee M.-K., Kim S.H. (2016). Development of paclitaxel-loaded liposomal nanocarrier stabilized by triglyceride incorporation. Int. J. Nanomed..

[41] Wang F., Pu C., Liu M., Li R., Sun Y., Tang W., Sun Q., Tian Q. (2022). Fabrication and characterization of walnut peptides-loaded proliposomes with three lyoprotectants: Environmental stabilities and antioxidant/antibacterial activities. Food Chem..

[42] Wang M., Zhao T., Liu Y., Wang Q., Xing S., Li L., Wang L., Liu L., Gao D. (2017). Ursolic acid liposomes with chitosan modification: Promising antitumor drug delivery and efficacy. Mater. Sci. Eng. C.

[43] Hao G., Xu Z.P., Li L. (2018). Manipulating extracellular tumour pH: An effective target for cancer therapy. Rsc. Adv..

[44] Tian L., Bae Y.H. (2012). Cancer nanomedicines targeting tumor extracellular pH. Colloid Surf. B Biointerfaces.

[45] Chu S.L., Shi X.L. (2022). pH-Responsive Polymer Nanomaterials for Tumor Therapy. Front. Oncol..

[46] Vander Heiden M.G., Cantley L.C., Thompson C.B. (2009). Understanding the Warburg Effect: The Metabolic Requirements of Cell Proliferation. Science.

[47] Tan C., Xue J., Lou X., Abbas S., Guan Y., Feng B., Zhang X., Xia S. (2014). Liposomes as delivery systems for carotenoids: Comparative studies of loading ability, storage stability and in vitro release. Food Funct..

[48] Yang X.-Y., Li Y.-X., Li M., Zhang L., Feng L.-X., Zhang N. (2013). Hyaluronic acid-coated nanostructured lipid carriers for targeting paclitaxel to cancer. Cancer Lett..

[49] Wang J., Ma W., Guo Q., Li Y., Hu Z., Zhu Z., Wang X., Zhao Y., Chai X., Tu P. (2016). The effect of dual-functional hyaluronic acid-vitamin E succinate micelles on targeting delivery of doxorubicin. Int. J. Nanomed..

[50] Cannito S., Bincoletto V., Turato C., Pontisso P., Scupoli M.T., Ailuno G., Andreana I., Stella B., Arpicco S., Bocca C. (2022). Hyaluronated and PEGylated Liposomes as a Potential Drug-Delivery Strategy to Specifically Target Liver Cancer and Inflammatory Cells. Molecules.

[51] Lee J.A., Spidlen J., Boyce K., Cai J., Crosbie N., Dalphin M., Furlong J., Gasparetto M., Goldberg M., Goralczyk E.M. (2008). MIFlowCyt: The minimum information about a Flow Cytometry Experiment. Cytometry A.

[52] Hudiyanti D., Al Khafiz M.F., Anam K., Siahaan P., Suyati L. (2021). Assessing encapsulation of curcumin in cocoliposome: In vitro study. Open Chem..

[53] Ravar F., Saadat E., Gholami M., Dehghankelishadi P., Mahdavi M., Azami S., Dorkoosh F.A. (2016). Hyaluronic acid-coated liposomes for targeted delivery of paclitaxel, in-vitro characterization and in-vivo evaluation. J. Control. Release.

[54] Kim E., Yang J., Park J., Kim S., Kim N.H., Yook J.I., Suh J.-S., Haam S., Huh Y.-M. (2012). Consecutive Targetable Smart Nanoprobe for Molecular Recognition of Cytoplasmic microRNA in Metastatic Breast Cancer. ACS Nano.

[55] Nascimento T.L., Hillaireau H., Vergnaud J., Fattal E. (2016). Lipid-based nanosystems for CD44 targeting in cancer treatment: Recent significant advances, ongoing challenges and unmet needs. Nanomedicine.

[56] Dekker E., Tanis P.J., Vleugels J.L.A., Kasi P.M., Wallace M.B. (2019). Colorectal cancer. Lancet.

[57] Bray F., Ferlay J., Soerjomataram I., Siegel R.L., Torre L.A., Jemal A. (2018). Global cancer statistics 2018: GLOBOCAN estimates of incidence and mortality worldwide for 36 cancers in 185 countries. CA Cancer J. Clin..

[58] Guren M.G. (2019). The global challenge of colorectal cancer. Lancet Gastroenterol. Hepatol..

[59] Siegel R.L., Miller K.D., Sauer A.G., Fedewa S.A., Butterly L.F., Anderson J.C., Cercek A., Smith R.A., Jemal A. (2020). Colorectal cancer statistics, 2020. CA Cancer J. Clin..

[60] Siegel R.L., Miller K.D., Jemal A. (2019). Cancer statistics, 2019. CA Cancer J. Clin..

[61] Chhikara B., Parang K. (2022). Global Cancer Statistics 2022: The Trends Projection Analysis. Chem. Biol. Lett..

[62] Spandana K.M.A., Bhaskaran M., Karri V., Natarajan J. (2020). A comprehensive review of nano drug delivery system in the treatment of CNS disorders. J. Drug Deliv. Sci. Technol..

[63] Kulkarni S.A., Feng S.-S. (2013). Effects of Particle Size and Surface Modification on Cellular Uptake and Biodistribution of Polymeric Nanoparticles for Drug Delivery. Pharm. Res..

[64] Xu Z., Gu W., Huang J., Sui H., Zhou Z., Yang Y., Yan Z., Li Y. (2005). In vitro and in vivo evaluation of actively targetable nanoparticles for paclitaxel delivery. Int. J. Pharm..

[65] Chaudhry G.-E., Akim A., Zafar M.N., Safdar N., Sung Y.Y., Muhammad T.S.T. (2021). Understanding Hyaluronan Receptor (CD44) Interaction, HA-CD44 Activated Potential Targets in Cancer Therapeutics. Adv. Pharm. Bull..

[66] Chen K.-L., Pan F., Jiang H., Chen J.-F., Pei L., Xie F.-W., Liang H.-J. (2011). Highly enriched CD133^+^CD44^+^ stem-like cells with CD133^+^CD44^high^ metastatic subset in HCT116 colon cancer cells. Clin. Exp. Metastasis.

[67] Takikawa M., Fujisawa M., Yoshino K., Takeoka S. (2020). Intracellular Distribution of Lipids and Encapsulated Model Drugs from Cationic Liposomes with Different Uptake Pathways. Int. J. Nanomed..

