# Supplementary material for: Multifunctional Liposomes Co-Modified with Ginsenoside Compound K and Hyaluronic Acid for Tumor-Targeted Therapy
Source: Polymers (Basel). 2024 Feb 1;16(3):405. doi: 10.3390/polym16030405 (PMC10857112; doi:10.3390/polym16030405)
Supplement: Supplementary file 1 [file polymers-16-00405-s001.zip › polymers-2803758-supplementary.pdf]

Supplementary Materials

Multifunctional Liposomes Co-Modified with Ginsenoside Compound K and Hyaluronic Acid for Tumor-Targeted Therapy

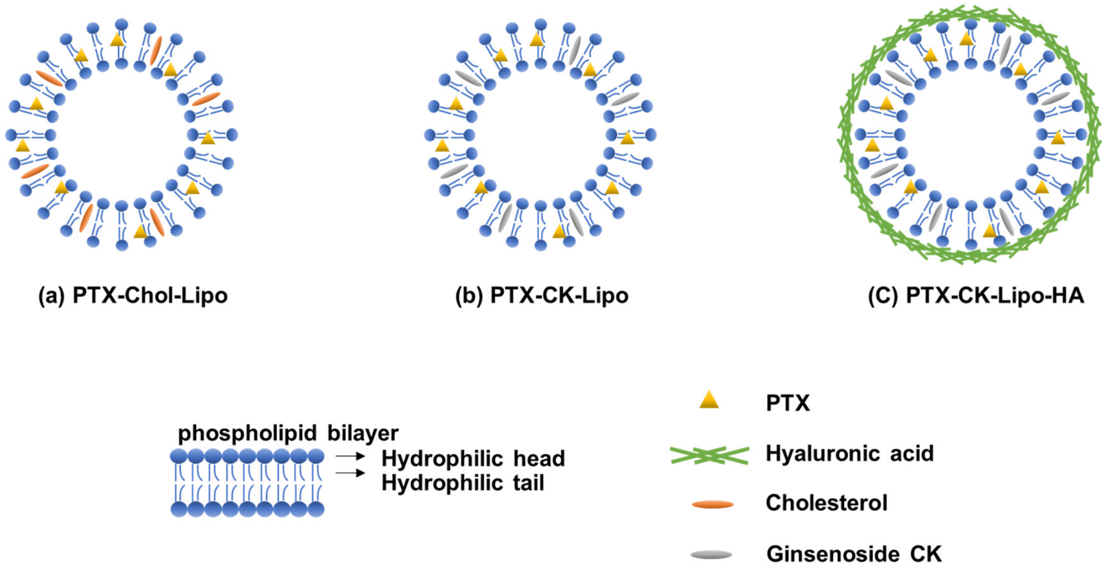

Figure S1. Basic structure of a liposome (a) P-Ch-Lip, (b) P-CK-Lip, and (c) P-CK-Lip-HA.

Project info 1:

To calculate the IC50 value, please follow these steps in GraphPad Prism:

1. Import the data for the cell inhibition rate into GraphPad Prism.
2. Navigate to the “Analyze” menu.
3. Choose the “Transform” option, followed by “Transform X into log(X)” to apply a logarithmic transformation to the X values.
4. Return to the “Analyze” menu.
5. Perform a double-click on “Nonlinear regression (curve fit)” to initiate the regression analysis.
6. From the available options, select “Dose response (inhibition)” as the analysis type.
7. To complete the process, click on “log (inhibitor) vs. normalized response-variable slop”.
